# Torque Teno virus DNA is found in the intracranial aneurysm wall—Is there a causative role?

**DOI:** 10.3389/fmed.2023.1047310

**Published:** 2023-01-19

**Authors:** Nícollas Nunes Rabelo, Marcia Harumy Yoshikawa, João Paulo Mota Telles, Giselle Coelho, Caio Santos de Souza, Natan Ponzoni Galvani de Oliveira, Tania Regina Tozetto Mendoza, Paulo Henrique Braz-Silva, Antonio Luiz Boechat, Manoel Jacobsen Teixeira, Eberval Gadelha Figueiredo

**Affiliations:** ^1^Department of Neurosurgery, University of São Paulo, São Paulo, SP, Brazil; ^2^Laboratory of Virology (LIM-52), University of São Paulo, São Paulo, SP, Brazil; ^3^Department of Stomatology, University of São Paulo, São Paulo, SP, Brazil; ^4^Department of Parasitology, Federal University of Amazonas, Manaus, AM, Brazil

**Keywords:** TTV, intracranial aneurysm, brain aneurysm, virus, molecular virus DNA, biomarker

## Abstract

**Objective:**

Torque Teno virus (TTV) is a recently discovered virus with high prevalence worldwide, that has been associated with vascular diseases. The aim of this study is to investigate the prevalence of TTV molecular DNA in the intracranial aneurysm (IA) artery walls.

**Method:**

Samples of IA walls were collected after microsurgical clipping from 35 patients with IA (22 ruptured/13 unruptured cases). The samples were submitted to molecular DNA extraction using the *EasyMag* automatized extractor and performed with *Qiagen DNA* extraction *Minikit* 250. The samples underwent PCR examination with primers for β-globin as internal control using the *Nanodrop*^®^ 2000 spectrophotometer. A quantitative (real-time) PCR with TTV-specific primers was performed. Clinical and radiological data of patients included was collected.

**Results:**

TTV was detected in 15 (42.85%) cases, being 10 (45.4%) ruptured and 5 (38.4%) unruptured (*p* = 0.732) lesions. Multiple IAs accounted for 14 (40%) cases. Five cases (17.2%) had TTV+ and multiple aneurysms (*p* = 0.73). Association between presence of virus and aneurysm rupture was not statistically significant (*p* = 0.96).

**Conclusion:**

This study demonstrated a relatively high prevalence of viral DNA in the walls of IAs. This is the first study to identify the presence of TTV DNA in IA’s samples, which was found more often in ruptured lesions. This is an exploratory study, therefore, larger studies are required to clarify the relationships between inflammation, viral infection, IA formation and rupture.

## Introduction

Recently, several studies have found an association between vascular diseases, including intracranial aneurysms (IA) and inflammation (1–9). However, the mechanisms of IA formation and rupture are not fully understood, and the potential triggers of inflammatory reaction have not been known thus far. In addition, IA pathogenesis may be different in distinct clinical settings as the well-known risk factors, e.g., arterial hypertension, familiar history and smoke, does not explain this phenomenon in all cases (10).

Chronic local inflammation may cause lesion of the arterial vessel, accompanied by changes in its layers secondary to inflammatory processes, including infiltration of T cells and leukocytes, complement activation, modification of tight junctions, and release of interleukins by immune cells. This mechanism leads to formation of an IA and eventually contributes to its rupture (10).

Conversely, virus is a well-studied risk factor for acute and chronic inflammation in human tissues. Pathogens, including viruses, have previously been studied and detected in other conditions, such as in atherosclerotic plaques and abdominal aortic aneurysms, corroborating the relationship between chronic infections and the development of vascular lesions (1, 11). However, the role of viral infection on the inflammation of the intracranial aneurysmal wall has not been investigated yet.

Torque Teno virus (TTV from Latin *torques* and *tenuis*, meaning “necklace” and “thin,” respectively) has been recently recognized as a highly prevalent virus that indolently infects human tissues (12). The prevalence of TTV infection worldwide is extremely high, reaching 95% and is dissociated from age, health conditions and socio-economic standings (13). TTV infection usually does not induce any clear clinical manifestation but may be occasionally associated with disorders in many organs (14). Analysis *in vivo* revealed the tropism of TTV for lymphoid cells, including T- and B-lymphocytes, monocytes, natural killer (NK) cells, granulocytes and other polymorphonuclear cells (15–17).

The development of new techniques devised to analyze the viral genome has made it possible to sequence molecular DNA and increase understanding about the impact of the presence of the virus on the vessel wall on the pathophysiology of vascular diseases (18). New viruses and their tissue distribution in the body has been better understood, quickly changing previous concepts concerning the complexity of those pathogens and their interactions with human’s tissues (19).

In this scenario, viral detection in the IA wall may support the hypothesis that the presence of virus in vascular tissues might be an additional risk factor to IA formation and rupture process, due to the breakdown of its layers through an inflammatory process. Therefore, the aim of this study was to investigate the prevalence of DNA of TTV in the wall of IAs and discuss its potential influence on IA pathogenesis.

## Materials and methods

### Ethical standards

This research project was approved by the *Ethics and Research Committee of Hospital das Clinicas*, FMUSP (HCFMUSP) (Online registration CAPPesq: 15226 approved 06/20/2016) and on the *Brazil Platform* of the Misnitry of Health (CAAE number: 61719416.6.0000.0068). This study was performed in the Central Institute of HCFMUSP and in the Laboratory of Virology (LIM-52), from the Institute of Tropical Medicine of São Paulo (IMT).

### Population data

**FIGURE 1:**
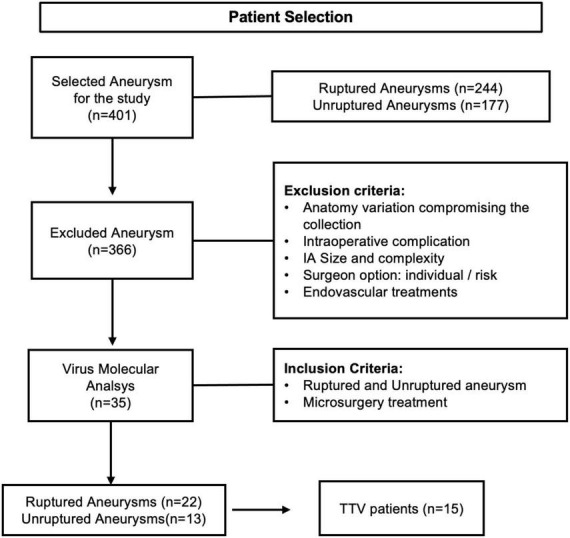
Flowchart of patient selection. Inclusion and exclusion criteria and patients selected.

From January 2018 to November 2019, 401 patients were admitted with IAs in the HCFMUSP. 244 patients presented ruptured and 177 unruptured aneurysms. 366 cases were excluded after applying the inclusion and exclusion criteria. This study comprised 35 IAs samples of aneurysm wall collected during open surgical procedures (Figure 1).

### Inclusion criteria

Patients admitted in the Emergency Department with diagnosis of ruptured aneurysm or surgical elective patients diagnosed with unruptured aneurysm were included if microsurgical treatment was carried out.

### Exclusion criteria

Patients submitted to endovascular treatment or those whose severity of the clinical picture precluded any treatment were excluded. Anatomical variations and technical difficulties compromising the sample extraction, intraoperative complications or high risk of aneurysm bleeding during surgery, IA size, location, and complexity, surgeon’s experience and discretion were other factors considered to exclude patients from the study.

### Intraoperative sample extraction

**FIGURE 2:**
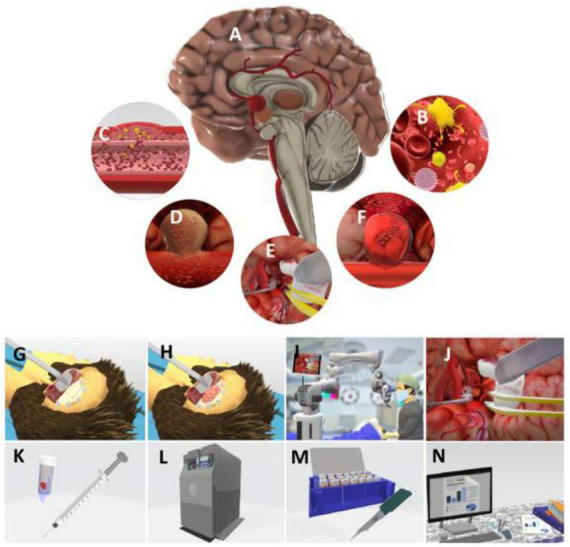
Schematic sequence of the study method. Panel **(A)** represents the intracranial aneurysm; **(B)** pathogenic oral virus that promote inflammatory changes in the microbiota in specific host; **(C)** virus can move to the blood flow and deposit in cerebral vessels, inducing inflammatory response and changes in vessel wall, which added to risk factors already known can induce the formation of an aneurysm; **(D)** unruptured cerebral aneurysm; **(E)** microsurgery for Aneurysm; **(F)** ruptured cerebral aneurysm—arterial rupture and consequent blood leakage into the subarachnoid space. **(I)** subarachnoid hemorrhage; **(G,H)** steps of the cerebral microsurgery—primarily the brain access is planned, then the craniotomy is performed, followed by dural opening and dissection of brain cisterns; **(I)** microsurgery being performed, using vascular techniques, Microscope Vario—ZEISS^®^ and microsurgical instrumental to manage the cerebral aneurysm; **(J,K)** aneurysm clipping—a tissue sample from the aneurysm domus is collected and preserved in an Eppendorf tube with 3 ml of saline solution 0.9%; **(L)** during the period between the sample removal and the final transportation, the material is preserved in a freezer at –20°C; **(M,N)** in the IMT laboratory, the tissue is preserved at –80°C, then microbiological and molecular analysis are performed.

**FIGURE 3:**
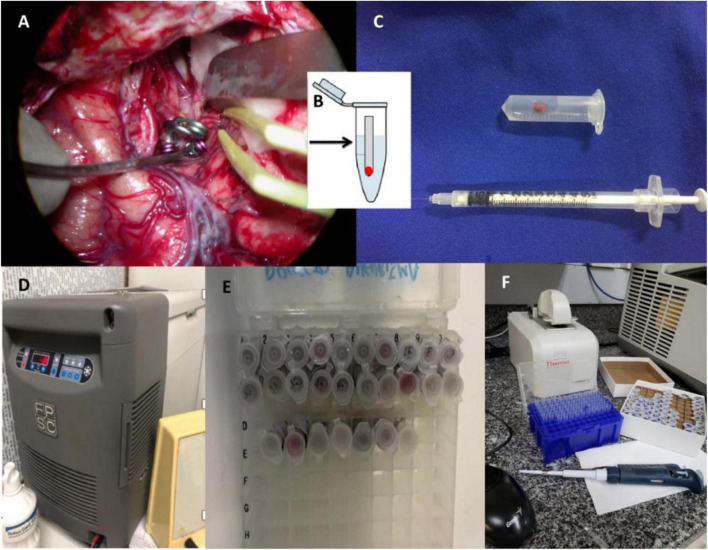
Sample collection protocol. **(A)** Figure referring to an example case of vascular microsurgery. This figure shows the clipped middle cerebral artery aneurysm with completed surgical hemostasis. At this point, the aneurysm vessel is prepared for collection, respecting the inclusion and exclusion criteria; **(B)** the sample is kept in Eppendorf sterile, with 0.9% saline solution; **(C)** the saline solution is quantified with a 3 ml syringe; **(D)** temporarily stored in a freezer at –20°C until the end of the day’s surgical activities. After collection, all samples were sent to the Virology Laboratory—Lim 52 of the Institute and stored at –80°C; **(E)** workstation; **(F)** preparation and extraction of DNA in viral PCR.

Thirty-five samples of aneurysm wall were collected in the operating room, including 22 ruptured and 13 unruptured aneurysms. The samples were collected after microsurgical clipping and intraoperative hemostasis. The tissue samples were temporarily stored in a freezer at −20°C until the completion of the surgical activities of the day, and then transferred to the Laboratory of Virology of the IMT, where they were stored at –80°Celsius. The Laboratory of Virology IMT was responsible for the appropriate management and microbiological analysis of the samples (Figures 2, 3).

### Microbiological analysis

Aneurysms’ tissue was submitted to molecular DNA extraction using the *EasyMag* automatized extractor (*NucliSENS*^®^ *easyMag*^®^ *bioMérieux*), according to the manufacturer’s instructions. All samples were suitable for DNA amplification evaluated by internal control (RNAse P). The DNA extraction was performed with *Qiagen DNA extraction Minikit* 250 (Qiagen, Germany, Hilden), following the instructions given by the manufacturer. The material extracted was submitted to PCR examination with primers for β-globin as internal control in order to evaluate the effectiveness of DNA extraction through the amplification process. This process was performed using the Nanodrop^®^ 2000 spectrophotometer (Thermo Fisher, MA, United States).

In order to verify the amount of viral DNA, the samples were analyzed by spectrophotometry, using the *Nanodrop*^®^ 2000 spectrophotometer (Thermo Fisher, MA, United States). During the analysis, a synthetic standard curve with known amounts of the synthetic oligonucleotide was used to quantify TTV.

### TTV PCR real time

A quantitative (real-time) PCR (qPCR) with TTV-specific primers and probe was performed using a synthetic standard curve with known amounts of oligonucleotides for TTV quantification (20). PCR real time was performed using *TaqMan Universal Master Mix* (ThermoFisher^®^) and according to the manufacturer’s instructions. The data was analyzed using *QuantStudio Design* & *Analysis Software* v.1.4.1. The detection limit for the test was from 1,34 log_10_ copies per reaction (analytical sensitivity (LOD > 95%) of 21.9 copies per reaction). Supplementary materials 1 and 2 show the characteristics of patients and viral load, and the sequence of primers used in the conventional PCR reaction for TTV.

### Statistical analysis

Descriptive statistics were applied to describe clinical and demographic characteristics of the patients. Continuous variables were described as mean (standard deviation) or median (interquartile distance), as appropriate under normal data analysis. Categorical data were described as frequencies (valid percentage). To compare means, a t-Student or Mann–Whitney test was applied, as appropriate, for continuous variables. *Chi-square* test for dichotomous variables. A two-tailed alpha level of 5% was adopted. The analyses were performed using the *GraphPad Prism* 8.0.0 software (San Diego, CA, USA).

## Results

Thirty-five cases of ruptured (*n* = 22; 62.9%) and unruptured (*n* = 13; 37.1%) aneurysms were analyzed. The study population’s mean age was 57.63 ± 1.87, while the mean age of patients with ruptured and unruptured IA was 56.8 ± 2.41 and 58.92 ± 3.00, respectively. Multiple aneurysms accounted for 14 (40%) of the sample, 7 patients being equally distributed between the ruptured and unruptured groups. The female gender represented most of the sample (*n* = 29; 82.9%), including 18 (62%) ruptured and 11 (38%) unruptured IAs. Male patients (*n* = 6; 17.1%) accounted for 4 (66.7%) ruptured and 2 (33.3%) unruptured lesions. Regarding the comorbidities of the study population, 23 patients had hypertension (65.7%), 1 had diabetes (2.8%) and 1 was on anticoagulants (2.8%). Only 1 (2.8%) subject had an immunodeficiency status – HIV infection. TTV was detected in 15 (42.9%) of the 35 samples, being 10 (66.7%) from ruptured and 5 (33.3%) from unruptured aneurysms (Table 1).

**TABLE 1:** (A) Demographic characteristics of 35 patients with aneurysm; (B) patient characteristics categorized by gender and virus positivity on the aneurysm wall and its relationship to multiple aneurysms. X^2^ test = 1.83; p = 0.4; (C) distribution of risk factors for aneurysm formation and rupture among patients TTV positive and TTV negative.

A			
	Patients	Ruptured aneurysm *N* (%)	Unruptured aneurysm
	*N* (%)		*N* (%)
Total	35 (100)	22 (100)	13 (100)
TTV	15 (42.8)	10 (45.4)	5 (38.4)
Age	57.63 ± 1.87	56.8 ± 2.41	58.92 ± 3.00
Female, *N* (%)	29 (82.8)	18 (81.8)	11 (84.6)
Multiple aneurysms	14 (40)	7 (31.8)	7(53.8)
Hypertension	23 (65.7)	15 (68.1)	8 (61.5)
Diabetes	1 (2.8)	1 (4.5)	0 (0)
Immunodeficiency^a^	1 (2.8)	1 (4.5)	0 (0)
Anti-coagulation	1 (2.8)	1 (4.5)	0 (0)
Other comorbidities^b^	7 (20)	1 (4.5)	6 (46.1)
Smoking	18 (51.4)	13 (59)	5 (38.4)
Alcohol abuse	2 (5.7)	2 (9)	0 (0)
**B**			
	**Patients** ***N* (%)**	**TTV** ***N* (%)**	**Multiple aneurysms *N* (%)**
Total	35 (100)	15 (100)	14 (100)
Female	29 (82.9)	11 (73.3)	13 (92.9)
Male	6 (17.1)	4 (26.7)	1 (7.1)
**C**			
**Risk factor**	**TTV +** ***N* (%)**		**TTV –** ***N* (%)**
Total	15 (100)		20 (100)
Hypertension	9 (60)		14 (70)
Diabetes	0 (0)		1 (5)
Immunodeficiency	1 (6.6)		0 (0)
Anti-coagulation	0 (0)		1 (5)
Smoking	8 (53.3)		10 (50)
Alcohol abuse	1 (6.6)		1 (5)
Previous HSA	1 (6.6)		0 (0)

^a^Patient living with HIV.

^b^Included patients with epilepsy (*n* = 1), hypothyroidism (*n* = 1), psychiatric disorders (*n* = 3), chronic renal disease (*n* = 1) and cardiomyopathy (*n* = 1).

In the female population (*n* = 29), multiple aneurysms accounted for 13 (44.8% of total female population; 92.8% of the total multiple cases) and presence of TTV for 11 cases (37.9% of total female population; 73.4% of total cases of TTV). In male population, presence of TTV was verified in 4 cases (66.7% of total male population; 26.7% of total cases of TTV). The association of both gender and presence of multiple aneurysms with detection of viral DNA were not statistically significant (*x*^2^ = 1.83; *p* = 0.4) (Table 1). TTV and multiple aneurysms were associated in 5 patients (33.3% of total cases of TTV). Among the 21 (60% of total population) patients without multiple aneurysms, 10 had positive TTV (47.6% of non-multiple cases) in aneurysm sample. The association between multiple aneurysms and TTV was not statistically significant (*x*^2^ = 0.12; *p* = 0.73) (Table 2).

**TABLE 2:** (White color line) TTV+ frequency in patients with ruptured aneurysms.

	TTV + *N* (%)	TTV – *N* (%)	Total *N* (%)
Total	15 (42.8)	20 (57.1)	35 (100)
Ruptured aneurysm	10 (45.5)	12 (54.5)	22 (100)
Unruptured aneurysm	5 (38.5)	8 (61.5)	13 (100)
One IA	10 (47.6)	11 (52.4)	21 (100)
Multiple IA	5 (35.7)	9 (64.3)	14 (100)

*X*^2^ test = 0.003, *p* = 0.96. (Gray color line) Association between multiple aneurysms and presence of TTV. *N*, number of patients; IA, intracranial aneurysm. *x*^2^ = 0.12, *p* = 0.73.

The IA characteristics are shown in Table 3. Most aneurysms were located at the middle cerebral artery (MCA; *n* = 18), mirror multiple aneurysms were present in 10 cases of MCA lesions. There was no ruptured aneurysm in pericallosal and ophthalmic arteries. No aneurysms at PCoA and PICA accounted for multiple cases. MCA aneurysms comprise the majority of ruptured (*n* = 12; 54.5%) and unruptured (*n* = 6; 46.1%) cases; MCA was the most frequent IA location of patients with multiple aneurysms (*n* = 8; 57.1%), and positive TTV (*n* = 7; 46.7%) cases. However, location was not associated with presence of TTV.

**TABLE 3:** Aneurysm characteristics.

	Patients *N* (%)	TTV *N* (%)	Ruptured aneurysm *N* (%)	Unruptured aneurysm *N* (%)	Multiple aneurysms *N* (%)
MCA	18 (51.4)	7 (43.7)	12 (54.5)	6 (46.1)	8 (57.1)
ACoA	6 (17.1)	2 12.5)	4 (18.1)	2 (15.3)	2 (14.2)
Pericallosal	3 (8.5)	2 (12.5)	0 (0)	3 (23)	1 (7.1)
ICA	3 (8.5)	2 (12.5)	2 (9)	1 (7.69)	1 (7.1)
ACoP	2 (5.7)	0 (0)	2 (9)	0 (0)	0 (0)
PICA	1 (2.8)	1 (6.2)	1 (4.5)	0 (0)	0 (0)
Ophthalmic	1 (2.8)	1 (6.2)	0 (0)	1 (7.7)	1 (7.1)
ACA	1 (2.8)	0 (0)	1 (4.5)	0 (0)	1 (7.1)
Total	35 (100)	15 (100)	22 (62.9)	13 (37.1)	14 (40)

N, number of patients; MCA, middle cerebral artery; ACoA, anterior communicating artery; ICA, internal carotid artery; ACoP, posterior communicating artery; PICA, posterior inferior cerebellar artery; ACA, anterior cerebral artery.

TTV DNA was found in 10 (45.4%) ruptured and in 5 (38.4%) unruptured cases. Although there was higher prevalence of viral presence in ruptured cases, the difference was not statistically significant between groups (*x*^2^ = 0.003; *p* = 0.96). The association between the presence of TTV and mortality was not statistically significant as well (*x*^2^ = 0.18; *p* = 0.67) (Table 2).

## Discussion

The Torque Teno virus is a virus with a single stranded, circular, negative sense DNA genome, containing 3.8 kb. It was first isolated in 1997 from a patient with posttransfusion hepatitis of unknown etiology and it is the prototype of a new virus family (21). The TTV is grouped into the Anelloviridae family, genus *Alphatorquevirus*, and was the first virus discovered from this family able to infect humans (12). The TTV infection does not induce any distinctive clinical manifestation, but it is associated with some disorders (14). It was raised the hypothesis that TTV was an agent that induces posttransfusion non-A hepatitis, for example. Other studies demonstrated that this virus is far more ubiquitous in the human organism, and analysis *in vivo* revealed the tropism of TTV for lymphoid cell, including T- and B-lymphocytes, monocytes, natural killer (NK) cells, granulocytes and other polymorphonuclear cells (15–17). However, the receptor used for invasion is still unknown (13).

The prevalence of TTV infection in the population worldwide has been found to be extremely high, reaching 95% and is dissociated from age, health conditions and socio-economic conditions (13). The geographical distribution of different genotypes of TTV is not homogeneous, but some of them are globally distributed, reaching even human populations with little or no contact with others, as the undisturbed indigenous tribes from Papua New Guinea, in Oceania (22).

The infection caused by different species of this virus is acquired early in life through several routes, including transfusion, respiratory, oral-fecal, and sexual (23). TTV viremia is extremely frequent in the general population, making the transfusion of contaminated blood products an important way of transmission (24).

Currently, TTV has been considered a potential biomarker of human immunocompetence, since there is evidence that TTV viremia is inversely related to percentage of T lymphocytes. In patients with lymphoma and myeloma, the decrease of CD8 + cells following the chemotherapy was concomitant to the increase of TTV levels (25), whereas patients with low CD4 + cell count demonstrated TTV viremia significantly elevated (26). Other medical conditions that affect the immune system response are also associated with peaks of TTV viremia, including sepsis, untreated solid cancer and stem cell and solid organs transplantation (16, 27–29).

The TTV infection induces lifelong viremia and, consequently, the virus can be identified in several organs - such as liver, lung, bone marrow, spleen, and other lymphoid tissues (30). The diagnosis is made with the detection of viral DNA in plasma or other biological samples. The high prevalence and the chronic infection suggest that TTV established a successful interaction with the host organism, by evading the immune response. It has been proposed that the DNA encoded in the TTV genome are used to target transcripts of the host cell, including a cofactor called NMI (N-myc-interactor), resulting in the inhibition of interferon signaling and consequent impairment of the host response (31).

Even though the chronic infection does not have any clear clinical manifestation, it is potentially associated with cardiovascular diseases, having already demonstrated its direct involvement in the formation of atheromatous plaques in cardiac patients (32). Its mechanism is associated with the interference in the activity of nuclear factor kappa beta (NF κB), an important transcription factor for inflammation and immunomodulation. It is also possible that induction of Th2 response during the viral replication is included in that process (32).

TTV is also able to change the metabolism of the host cell using proteases (33). After infecting cells of the immune system, such as macrophages and T cells, TTV could interfere in the processing and presentation of antigens, expressing new epitopes that would serve as triggers for autoimmunity. It could also, as Cytomegalovirus (CMV) (34), interfere with the expression of MHC class I molecules.

### TTV and IAs

Chronic local inflammation may lead to weakening of the arterial wall and, consequently, formation of an IA and rupture (10). This condition, when associated with known risk factors, may play a role in the formation of cerebral aneurysm. Pathogens, including viruses, have previously been studied and detected in other conditions, such as in atherosclerotic plaques and abdominal aortic aneurysms, corroborating the hypothesis of a relationship between chronic infections and the development of vascular changes (1, 11).

Thus far, there is no study in the literature that analyzes the presence of virus in samples of IAs (3, 6). Previous studies also reported presence of CMV in the abdominal aortic aneurysms (11). CMV is associated with vascular diseases, including aortic aneurysms. CMV usually establishes latency after the primary infection and a subclinical infection. However, it can also interfere with cellular function in CMV-infected SMC, macrophages, and EC, contributing to the pathogenesis of vascular lesions (17). No similar investigation has studied the presence of virus in the wall of IAs. Therefore, this study is unprecedented regarding the analysis of viruses in IAs.

It is important to notice that the influence of the TTV infection in the aneurysm pathogenesis may be different from the mechanism proposed for mycotic aneurysm, since the former is a silent and chronic process that can take years to occur and may interact with environmental and genetic factors, whereas the latter is a complication secondary to fungal and bacterial infections mostly associated with endocarditis (35).

In this study, TTV DNA was found in 15 out of 35 aneurysm wall samples, with similar distribution of risk factors and comorbidities between the group with positive and the group with negative TTV results. Additionally, although serum TTV is pointed out was a potential biomarker of immunocompetence (25) and that conditions of decreased immunocompetence were expected to present higher probability of viral infiltration in tissues, only one out 15 patients with TTV DNA in aneurysm sample had an immunodeficiency status.

Concerning the influence of TTV in the aneurysm rupture, although there was higher prevalence of viral presence among ruptured cases (45.4%) than among unruptured cases (38.4%), the difference was not statistically significant. This finding suggests that TTV may not be crucial for the rupture event, but other known risk factors such as hypertension, age, size and site of the aneurysm. Alternatively, it may reflect a statistical artifact due to the study’s limited sample size and, consequently, statistical power. Another study is currently being performed to increase the sample size and overcome this limitation. In both ruptured and unruptured groups arterial hypertension was highly prevalent, supporting the importance of shear stress on the vessel wall as a contributing factor for the formation of aneurysms.

It is important to highlight that many recent studies proposed that TTV sequential measurement by PCR DNA, in tissue samples or in saliva is potentially useful to assess patient inflammatory characteristics (36–38). High level of TTV viremia is related to the patient’s inflammatory state (36–38). Inflammatory activity, commonly found in IA wall, may be cause or consequence for the relatively high prevalence of TTV, particularly in ruptured lesions (36–39).

### Limitations

There are some limitations in this study. First, the interpretation of the results is limited by the sample size, justified by multiple factors, including eligibility to collect the material in the operating room, training of the surgical team to obtain the fragment, the risk of intraoperative complications, endovascular treatment, and clinical severity (which makes collection often impractical during surgery).

Losses of follow-up, problems with data consistency, impossibility of following long-distance patients and giving up the participation in the study, restricted the follow-up and limited the analysis of association between clinical outcomes and viral infection. Therefore, this data might be satisfactory to describe the virus prevalence but not to determine clinical correlations.

Further studies with larger samples are essential to determine the exact relationships between inflammation, viral infection, and IA formation. Clarifying this issue may increase knowledge about the condition and contribute to the development of new interventions and prevention of aneurysmal disease.

## Conclusion

This study demonstrated a relatively high prevalence of viral DNA in the walls of IAs. This is the first study to identify the presence of TTV DNA in IA’s samples, which was found more often in ruptured lesions. This is an exploratory study, therefore, larger studies are required to clarify the relationships between inflammation, viral infection, IA formation and rupture.

## Data availability statement

The raw data supporting the conclusions of this article will be made available by the authors, without undue reservation.

## Ethics statement

The studies involving human participants were reviewed and approved by Ethics and Research Committee of Hospital das Clinicas da FMUSP (HCFMUSP). The patients/participants provided their written informed consent to participate in this study.

## Author contributions

NR, PB-S, and EF: conceptualization and methodology. MY, JT, GC, CS, NO, and TM: formal analysis and investigation. NR and MY: writing—original draft preparation. MY, NR, JT, and EF: writing—review and editing. MT and EF: supervision. All authors contributed to the article and approved the submitted version.
