# Supplementary material for: Torque Teno virus DNA is found in the intracranial aneurysm wall—Is there a causative role?
Source: Front Med (Lausanne). 2023 Jan 19;10:1047310. doi: 10.3389/fmed.2023.1047310 (PMC9894622; doi:10.3389/fmed.2023.1047310)
Supplement: Supplementary file 1 [file Table_1.pdf]

Supplementary Material

Supplementary Table 1. **Characteristics of patients and viral load**. CT: cycle threshold; TTV: Torque Teno Virus

Patient	TTV (CT)/Viral load
**1**	-
**2**	37.529 / 1.1752
**3**	-
**4**	36.77 / 3.000
**5**	-
**6**	-
**7**	33.273 / 35.871
**8**	34.003 / 21.364
**9**	-
**10**	-
**11**	-
**12**	32.481 / 62.892
**13**	-
**14**	33.358 / 33.763
**15**	33.852/ 23.779
**16**	-
**17**	-
**18**	-
**19**	-
**20**	-
**21**	37.656 / 1.601
**22**	28.505 / 1055.051
**23**	-
**24**	31.407 / 134.719
**25**	-
**26**	-
**27**	38.263 / 1.0404
**28**	-
**29**	37.179/ 2.244
**30**	-
**31**	35.379/ 8.048
**32**	38.315 / 1.002
**33**	32.901 / 40
**34**	-
**35**	-
