# Supplementary material for: Torque Teno virus DNA is found in the intracranial aneurysm wall—Is there a causative role?
Source: Front Med (Lausanne). 2023 Jan 19;10:1047310. doi: 10.3389/fmed.2023.1047310 (PMC9894622; doi:10.3389/fmed.2023.1047310)
Supplement: Supplementary file 2 [file Table_2.pdf]

Supplementary Table 2. **Sequence of primers used in the conventional PCR reaction for TTV**

Primer	**Sequence 5’-3’**
Forward	GTGCCGIAGGTGAGTTTA
Reverse primer	AGCCCGGCCAGTCC
Probe	TCAAGGGGCAATTCGGGCT
Synthetic curve	TTCGTAGCCCGGCCAGTCCCGTATAGCCCGAATTGCCCCTTGAATGCGTTAAACTCACCAICGGCACCTGATA
